# The relationship between NLRP3 rs10159239 and Vaspin rs2236242 gene variants and obstructive sleep apnea

**DOI:** 10.48101/ujms.v126.7603

**Published:** 2021-06-02

**Authors:** Buğra Kerget, Ferhan Kerget, Çiğdem Yüce Kahraman, Alperen Aksakal, Ömer Araz

**Affiliations:** a Department of Pulmonary Diseases, Ataturk University School of Medicine, Erzurum, Turkey; b Department of Infection Diseases and Clinical Microbiology, Health Sciences University Erzurum Regional Education and Research Hospital, Erzurum, Turkey; c Department of Medical Genetics, Ataturk University School of Medicine, Erzurum, Turkey; d Department of Pulmonary Diseases, Health Sciences University Erzurum Regional Education and Research Hospital, Erzurum, Turkey

**Keywords:** Obstructive sleep apnea, polymorphism, vaspin, NLRP3, Elisa

## Abstract

**Background:**

In obstructive sleep apnea (OSA), recurrent upper airway obstruction and apnea/hypopnea episodes result in endothelial dysfunction, which leads to the release of many proinflammatory cytokines and reactive oxygen species (ROS). ROS induces NLRP3, a protein involved in the synthesis of interleukin (IL)-1 and IL-18; vaspin is a serine protease inhibitor that has an important role in suppressing the activation of NLRP3 inflammasome. In this study, we aimed to investigate the effect of NLRP3 rs10159239 (rs9239) and vaspin rs2236242 (rs6242) single nucleotide polymorphisms (SNPs) on OSA development.

**Methods:**

This study included 220 individuals who underwent polysomnography (118 patients with OSA and 102 healthy controls). NLRP3 rs9239 and vaspin rs6242 mutation frequencies were analyzed.

**Results:**

The NLRP3 rs9239 SNP genotype analysis revealed no statistically significant differences between the OSA and control groups. In the vaspin gene analysis, the rs6242 AA genotype was significantly more frequent in the OSA group compared with the control group, while the AT genotype was more frequent in controls (*P* = 0.004, *P* = 0.02). Comparison of rs6242 allele levels showed that the A allele was significantly more frequent in OSA patients than in controls (*P* = 0.03). The AA genotype was significantly more frequent in patients with severe OSA than in patients with mild or moderate OSA and the control group (*P* = 0.001 for all). Serum vaspin levels were significantly lower in carriers of the AA genotype than those with AT and TT genotypes (*P* = 0.001).

**Conclusion:**

The vaspin rs6242 SNP AA genotype increased susceptibility to OSA, while the AT genotype appeared to be protective. The lower plasma vaspin levels in OSA compared with the control group and in patients with the AA genotype suggest that vaspin may be a protective biomarker for OSA.

## Introduction

Obstructive sleep apnea (OSA) is characterized by recurrent episodes of apnea/hypopnea and frequent reductions in blood oxygen saturation resulting from upper airway obstruction (1). In OSA patients, repeated oxygen desaturations and subsequent endothelial dysfunction induce the release of strong proinflammatory biomarkers, particularly tumor necrosis factor alpha (TNF-α), interleukin-6 (IL-6), C-reactive protein (CRP), leptin, and reactive oxygen species (ROS) (2–4).

ROS play an important role in the exacerbation of inflammation in OSA and increase in correlation with disease severity (5). ROS are also important in the synthesis of Nod-like receptor protein 3 (NLRP-3) (6). NLRP3, adapter protein ASC-1, and caspase-3 precede the synthesis of IL-1 beta and IL-18, which are key mediators of inflammation. A study investigating NLRP3, IL-1 beta, and IL-18 levels in OSA patients showed that the levels of proinflammatory cytokines generated due to oxidative stress increased independently of NLRP3 synthesis (7). On the other hand, in studies on diabetes mellitus and coronary artery disease, which are frequent complications in OSA, NLRP3 levels were found to increase in correlation with disease severity (8,9). In patients with the NLRP3 rs10159239 (rs9239) A allele, the increased NLRP3 expression was associated with a significant increase in the severity of coronary artery disease (8).

The serine protease inhibitor vaspin, a novel adipocytokine synthesized by white adipose tissue, plays an important role primarily in the anti-inflammatory balance, as well as overcoming insulin resistance and preventing obesity (10,11). A comparative analysis of serum vaspin levels in OSA patients and a control group showed that patients with severe OSA had significantly lower serum vaspin levels compared with controls (12). Studies of vaspin rs2236242 (rs6242) single nucleotide polymorphism (SNP) demonstrated that individuals carrying the A allele had higher risk of developing type 2 diabetes mellitus (13). In another recent study, patients with the vaspin A allele were also found to be at higher risk of developing coronary artery disease (14). However, the serum vaspin level was not associated with genotype frequency in either of these studies.

The oxidative stress, endothelial dysfunction, and insulin resistance that occur as a result of recurrent apnea/hypopnea episodes in OSA predispose to many diseases, especially coronary artery disease and diabetes. As this study aimed to elucidate the role of NLRP3 rs9239 and vaspin rs6242 SNPs in the development of OSA, patients with comorbidities were not included in order to eliminate the confounding effect of comorbidities.

## Materials and methods

### Study design

This single-center case–control study included individuals aged 20 years and over who presented to the chest diseases department of our hospital with complaints of snoring, witnessed apnea, and/or excessive daytime sleepiness and underwent polysomnographic evaluation between August 2020 and April 2021. A written informed consent was obtained from all patients. This study was designed and conducted in accordance with the ethical guidelines set forth in the Declaration of Helsinki, and the study protocol was approved by the local ethics committee (B.30.2.ATA.0.01.00/63). This research was supported by the Ataturk University Scientific Research Project Office (project number 6607).

### Study population

A total of 243 adults who underwent polysomnography were screened for inclusion in the study. Exclusion criteria were defined as the presence of chronic or clinically significant infectious or inflammatory conditions in the past month, asthma, chronic obstructive pulmonary disease, malignancy, current or past smoking history, invasive surgical intervention in the past month, uncontrolled hypertension, high fasting blood glucose, diabetes, cerebrovascular disease, kidney disease, and coronary artery disease. According to these criteria, 1 patient with a history of upper respiratory tract disease in the last month, 12 patients in whom coronary artery disease was detected during follow-up, and 10 patients with high fasting blood glucose were excluded. As a result, a total of 220 patients were included in the study. Controls were matched with OSA patients by age and gender.

Based on the results of polysomnography, 118 patients with a apnea/hypopnea index (AHI) > 5 were regarded as having OSA and were classified by severity as mild (AHI: 5–15), moderate (AHI: 16–30), or severe (AHI: >30). The remaining 102 individuals who had AHI values < 5 were included in the control group.

### Polysomnography

All participants underwent full polysomnography monitoring using a Compumedics E-series Sleep System (Compumedics Sleep, Melbourne, Australia). Surface electrodes were used to record electroencephalography, right and left electrooculographies, and submental electromyography. Ventilatory airflow through the nose and/or mouth was measured, and inductive plethysmography bands were used to monitor body position and respiratory movements of the chest and abdomen. Arterial oxygen saturation was measured transcutaneously via finger-tip oximeter.

Apnea was defined as continuous cessation of airflow for ≥ 10 sec. Hypopnea was defined as at least 50% reduction in airflow for ≥ 10 sec together with oxygen desaturation of ≥3% or an arousal from sleep on EEG. Apneas were classified as obstructive, central, or mixed according to the standard American Academy of Sleep Medicine criteria (1).

### Molecular analysis

#### DNA isolation protocol

Blood samples were collected into ethylenediaminetetraacetic acid (EDTA) tubes. DNA of the patients was extracted using QIAamp DNA Mini Kit (Qiagen, Hilden, Germany) according to the manufacturer’s protocol. DNA quality was measured using Nanodrop (ND-1000, Thermo Fischer Scientific, Wilmington, DE, USA).

#### Analysis of rs10159239 and rs2236242 single‑nucleotide olymorphisms

Allele-specific SNPtype assays were run using the Fluidigm Flex Six™ Genotyping IFC (Fluidigm Corp., South San Francisco, CA, USA). Specific target amplification (STA) was performed to increase the number of molecular targets at the beginning. The determined thermal cycle program was run using the Bioer Gene Pro Thermal cycler (95°C for 15 min followed by 14 cycles of 95°C for 15 sec and 60°C for 4 min). SNPtype assay mixes and sample mixes were prepared according to the manufacturer’s protocol. After all the dynamic array was loaded with a 4 μL of each 10× assay mix and 5 μL of each sample mix. Dynamic array was placed on the IFC Controller HX (Fluidigm), and the loading process was completed. Then, dynamic array was placed on the BioMark system (Fluidigm), which performs the thermal cycling and fluorescent image acquisition. A data collection software was used on BioMark system. Genotyping application, ROX passive reference, and SNPtype-FAM and SNPtype-HEX probe types were selected. The SNPtype E FLEXsix v1 protocol was used for thermal cycling and image capture. At the end, we assessed the genotypes of the samples.

### Measurement of biochemical markers

Peripheral venous blood samples were collected after 15 min of rest into tubes containing EDTA. Vaspin levels were measured using the enzyme-linked immunosorbent assay (Elabscience Human ELISA kit, UK).

### Statistical analysis

The data were analyzed using IBM SPSS Statistics for Windows version 24.0 (IBM Corp., Armonk, NY). Comparisons of characteristics between cases and controls were analyzed by χ^2^ test for categorical variables and independent sample t‐test or Mann–Whitney U test for continuous variables, as appropriate. The difference in allele and genotype frequencies was compared between control and OSA using Pearson’s χ^2^ test. Statistical significance of the observed genotype frequencies was evaluated according to the Hardy–Weinberg rule and compared to the expected genotype frequencies. Independent-samples t test was used to compare demographic data and laboratory parameters between groups. A *P*-value less than 0.05 was considered statistically significant.

### Hardy–Weinberg equilibrium

The Hardy–Weinberg equilibrium (HWE) is a principle that states that genetic variation in a population will remain constant from one generation to the next in the absence of disruptive factors, such as non-random reproduction, mutations, and natural selection. Therefore, the HWE defines an idealized state, and genetic variations in nature can be defined as deviations from this equilibrium state (15).

For example, in HWE, there is (A) and (a)allele *p* = allele frequency of (A) and *q* = allele frequency of (a), the expected genotype frequency under HWE is *p*2 for the AA genotype, 2*pq* for the Aa genotype, and *q*2 for the aa genotype.

p2+ 2pq+ q2= 1

i = (total number of A allele)/2 × n

*q* = (total number of a allele)/2 × n

After these calculations, our expected and observed genotype and allele frequencies are compared by χ2 test. If there is no significant difference in expected and observed parameters, we could say there is HWE for our study group.

## Results

The mean age in the OSA and control groups was 48.2 ± 23.6 years and 47.4 ± 22.1 years, respectively. Of the 118 patients in the OSA group, 62 (52.5%) were men and 52 (47.5%) were women, while the 102 controls included 55 men (53.9%) and 47 women (46.1%). There was no statistically significant difference in age or sex between the OSA and control groups.

The OSA group showed significantly higher body mass index (BMI), AHI, triglycerides, and oxygen desaturation index (ODI) compared with the control group (*P* = 0.04, 0.001, 0.001, 0.001, respectively) (Table 1).

**Table 1:** Comparison of laboratory test results in patients with obstructive sleep apnea syndrome (OSA) and controls

	Control (*n* = 102) Mean ± SD	All OSA patients (*n* = 118) Mean ± SD	*P*
Age (years)	47.4 ± 22.1	48.2 ± 23.6	0.42
BMI (kg/m^2^)	24.9 ± 7.4	28.6 ± 7.6	**0.04**
AHI	3.1 ± 1.2	52.6 ± 31.8	**0.001**
REM AHI	3.1 ± 1.7	28.5 ± 24.2	**0.001**
Triglycerides (mg/dl)	116.1 ± 47.2	143.2 ± 41.1	**0.001**
HDL cholesterol (mg/dl)	41.1 ± 8.2	42.3 ± 11.1	0.12
LDL cholesterol (mg/dl)	120.2 ± 42.4	124.5 ± 47.9	0.16
Cholesterol (mg/dl)	181.4 ± 30.4	187.2 ± 35.1	0.23
ODI	1.6 ± 0.8	34.1 ± 10,4	**0.001**
Fasting blood glucose (mg/dl)	79.2 ± 15.6	82.5 ± 18.4	0.18
HgbA1c (%)	5.7 ± 1.4	6 ± 1.2	0.06
Vaspin (ng/ml)	1,89 ± 1,76	1,02 ± 0,74	**0.01**

BMI: Body mass index, AHI: Apnea/hypopnea index, HDL: High-density lipoprotein, LDL: Low-density lipoprotein, ODI: Oxygen desaturation index, REM: Rapid eye movement sleep.

Bold indicates statistically significant parameters.

The NLRP3 rs9239 SNP analysis revealed that there was no statistically significant difference between the OSA and control groups (χ2: 0.98, df: 2, p = 0.66). (Table 2).

**Table 2:** Comparison of NLRP3 rs9239 genotype numbers between OSA and control groups

	AG, *n* (%)	AA, *n* (%)	GG, *n* (%)	*P*
Control (*n* = 102)	68 (66.6)	11 (10.9)	23 (22.5)	0.66
OSA (*n* = 118)	82 (69.5)	12 (10.1)	24 (20.4)	

*χ*^2^: 0.98, df: 2, OSA:Obstructive sleep apnea, A: Adenine, G: Guanine.

The frequency of the AT genotype was higher in the control group than in the OSA group, while the AA genotype was higher in the OSA group (odds ratio [OR]: 0.445, 95% CI: 0.219–0.91, *P* = 0.02 and OR: 2.21; 95% CI: 1.46–2.68, *P* = 0.004, respectively) (Table 3). Obesity-adjusted analysis showed that the difference in AA genotype frequency persisted (OR: 1.96, 95% CI: 0.98–2.57, *P* = 0.03), but there was no statistically significant difference in the AT genotype (OR: 0.63, 95% CI: 0.315–1.22, *P* = 0.06). In the evaluation of allele frequency, it was also observed that the frequency of the A allele was higher in the OSA group than in the control group.

**Table 3:** Comparison of vaspin rs6242 genotype / allele numbers between OSA and control groups

	Control	OSA	OR (95% CI)	*P*	OR (95% CI)*	P*
TT (*n*)	7 (6.9%)	9 (7.6%)	–	–	–	–
AT (*n*)	61 (59.8%)	25 (21.2%)	0.445 (0.219–0.91)	**0.02**	0.63 (0.315–1.22)	0.06
AA (*n*)	34 (33.3%)	84 (71.2%)	2.21 (1.46–2.68)	**0.004**	1.96 (0.98–2.57)	**0.03**
Allele						
T (*n*)	75	43	–	–		
A (*n*)	129	193	0.581 (0.381–0.912)	**0.03**		

*OSA*: Obstructive sleep apnea, A: Adenine, T: Thymine, OR: odds ratio, CI: confidence interval.

* : Adjusted for obesity.

Bold indicates statistically significant parameters.

The vaspin rs6242 allele and genotype frequencies in the study groups and comparison with the HW equilibrium are shown in Table 4. Both the control and OSA groups showed statistically significant differences in HW equilibrium analysis (*P* = 0.016, *P* = 0.006).

**Table 4:** Vaspin rs6242 Allele/genotype frequencies and test of Hardy–Weinberg equilibrium

	Control		OSA	
f(A)	0.63		0.82	
f(T)	0.37		0.18	
–	O	E	O	E
TT	7	13.9	9	3.8
AT	61	47.5	25	34.8
AA	34	40.4	84	79.39
	*χ*^2^: 8.259, df: 2, *P* = 0.016		*χ*^2^: 10.142, df: 2, *P* = 0.006	

*OSA*: Obstructive sleep apnea, A: Adenine, T: Thymine; f: Observed frequency of each allele (T or A), O: Observed genotype numbers, E: Expected genotype numbers under a Hardy–Weinberg equilibrium assumption; *χ*^2^: Chi-square values.

**Figure 1:**
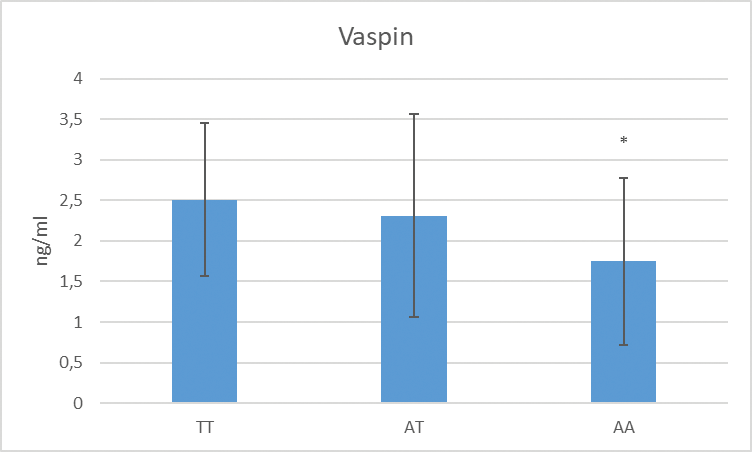
Evaluation of rs6242 genotype frequency and serum vaspin level. p*: Comparison of serum vaspin level in AA genotype with AT and TT genotype (*p* = 0.001).

The AA genotype was significantly more frequent in patients with severe OSA than in patients with mild or moderate OSA and the control group (*P* = 0.001 for all) (Table 5). The AT genotype did not differ in frequency based on OSA severity but was significantly more frequent in the control group (*P* = 0.001 for all). In the comparison of serum vaspin level in AA genotype with AT and TT genotype, a statistically significant higher was observed in AA genotype compared to AT and TT (*P* = 0.001 for all) (Figure 1).

**Table 5:** Comparison of genotype distributions in OSA patients according to disease severity

	Control (*n* = 102) *n* (%)	Mild OSA (*n* = 31) *n* (%)	Moderate OSA (*n* = 31) *n* (%)	Severe OSA (*n* = 56) *n* (%)	*P*
AT	61 (59.8)	13 (41.9)	7 (22.6)	5 (8.9)	0.001**
AA	34 (33.3)	11 (35.5)	19 (61.3)	47 (83.9)	0.001**
TT	7 (6.9)	7 (22.6)	5 (16.1)	4 (7.2)	> 0.05

P*: Comparison of AT genotype in control vs. other groups, P**: Comparison of AA genotype in severe OSA vs. other groups and in control vs. light OSA group; OSA: Obstructive sleep apnea, A: Adenine, T: Thymine.

Bold indicates statistically significant parameters.

## Discussion

No significant differences were observed between the OSA and control groups in the NLRP3 rs9239 genotype analysis. However, in the HWE analysis of vaspin rs6242 allele/genotype frequency, the AT genotype was more frequent than expected in the control group, and the AA genotype was more frequent in the OSA group. In our study excluding individuals with comorbidity, the AA genotype was observed to increase susceptibility to OSA compared to the control group, independently of BMI. Furthermore, comparisons based on OSA severity showed that the AA genotype was more frequent in patients with severe OSA as well. Individuals with the AT genotype had a lower risk of OSA. In the comparison of serum vaspin levels in the AA genotype group with the AT and TT genotypes, higher levels were observed in the AA genotype. The frequency of the A allele was higher in the OSA group than in the control group, suggesting that it may be an important risk factor for OSA susceptibility.

OSA is characterized by recurrent upper airway obstruction during sleep, increased respiratory effort in response to this obstruction, and frequent sleep interruptions (16). Successive episodes of apnea lead to increased sympathetic nervous system activity, oxidative stress, intrathoracic pressure fluctuations, sudden increases in systemic blood pressure, and hypoxia, all of which contribute to endothelial dysfunction (17–19).

Many cytokines such as TNF-alpha, IL-1, IL-2, IL-4, IL-6, IL-18, monocyte chemoattractant protein-1 (MCP-1), vimentin, and platelet-derived growth factor (PDGF) are produced by macrophages and T lymphocytes activated due to endothelial dysfunction and oxidative stress (20–22). Inflammatory cytokines contribute to a further progression of endothelial dysfunction and the development of comorbidities such as atherosclerosis and insulin resistance, which are commonly seen in OSA (21,23). It is worthy of note that continuous positive airway pressure (CPAP) therapy reduces levels of inflammatory cytokines in OSA patients (24).

Oxidative stress is known to be the main activator of NLRP3 synthesis, which, in turn, plays an important role in the synthesis of powerful proinflammatory cytokines, primarily IL-1 and IL-18 (25). In a study on patients with COPD, another condition in which oxidative stress is important, it was found that NLRP3 levels increased during acute exacerbations and decreased in correlation with clinical stability (26). Moreover, studies on patients with idiopathic pulmonary fibrosis with long-term hypoxia and accompanying comorbidities demonstrated that pirfenidone suppressed lung inflammation and fibrosis development by blocking the NLRP3 inflammasome (27). In studies investigating plasma NLRP3 levels in OSA patients, it was observed that the proinflammatory cytokine IL-1 and IL-18 levels increased independently of NLRP3 levels (7). Our literature search yielded few studies on the NLRP3 rs9239 SNP. In one study, a higher frequency of the A allele was associated with higher incidence of coronary artery disease and was also correlated with disease severity (8). Coronary artery disease was among the exclusion criteria for the patient and healthy control groups of our study. Detection of the AG allele in all 120 subjects in our study suggests that NLRP3 rs9239 SNP is not a risk factor associated with OSA development. Moreover, the absence of coronary artery disease in carriers of the A allele raises the need for more comprehensive studies evaluating the correlation between this SNP and coronary artery disease.

Research into the suppression of the strong proinflammatory effect of NLRP3 highlighted the activity of vaspin, an adipose tissue-derived serine protease inhibitor. Vaspin suppresses the activation of the NLRP3 inflammasome and also reduces IL-1 and TNF-alpha synthesis, in which NLRP3 plays an important role. In vitro studies demonstrated that vaspin also reduces the generation of mitochondrial free radicals (28). Analysis of plasma vaspin levels in OSA patients showed that after excluding those with diabetes mellitus and coronary artery disease, the vaspin level was lower among OSA patients than in controls (12). In another study, vaspin levels increased in correlation with BMI, waist circumference, waist/hip ratio, triglyceride level, and fasting blood glucose (28). In that study, which had no group homogenization in terms of parameters that may affect vaspin level, it was observed that vaspin increased in correlation with OSA severity and regressed with CPAP therapy. Studies on the vaspin rs2236242 SNP have shown that the frequency of type 2 diabetes mellitus was higher in individuals with the A allele than the control group (13). In a study of women in Upper Egypt, the A allele was found to be protective against obesity and diabetes mellitus, and carriers of this allele had lower plasma vaspin concentrations (29). In another study on its association with coronary artery disease, there was a significant correlation between the AT genotype and coronary artery disease, while the TT genotype was more frequent in controls. When compared in terms of plasma vaspin levels, the TT genotype had relatively higher vaspin levels than the AT genotype, but as there were only three patients with the AA genotype, the analysis was weak (14).

The OSA group in our study, which did not include patients with diseases associated with rs6242 SNP, such as coronary artery disease and diabetes mellitus, showed a higher frequency of the AA genotype compared with the control group. The AT genotype was detected at a higher rate in the control group compared with the OSA group, suggesting that this genotype may be protective against OSA. The statistically significant difference caused by increased AT genotype frequency in the control group and increased AA frequency in the OSA group observed in the HW equilibrium analysis also supported this. However, in the analysis adjusted for obesity and gender, this difference disappeared. When this is evaluated together with studies, suggesting that the A allele may be protective against obesity, it can be expected that BMI may be higher in those with the AT genotype compared to the AA genotype. Analysis of patients according to OSA severity showed that the AA genotype was more frequent in the severe group. In addition, serum vaspin levels were lower in individuals with the AA genotype compared with those with TT and AT genotype. This result can also be interpreted as evidence that vaspin, which has been reported as anti-inflammatory in studies conducted with OSA, is synthesized at lower levels in individuals with the A allele. This, in turn, may increase the susceptibility to OSA, in which impaired inflammatory/anti-inflammatory balance plays an important role.

Although patients with comorbidities were excluded in order to avoid their effect on NLRP2 rs9239 and vaspin rs6242 SNP genotype analysis, the most important limitation of our study was the inability to ensure BMI homogeneity between the OSA and control groups. This obscured the susceptibility and protective effect of the vaspin rs6242 SNP between the control and OSA groups. Large-scale studies with BMI-homogeneous samples are needed to further elucidate this relationship.

In summary, there was no difference in the OSA development according to NLRP3 level, whereas the OSA was significantly more prevalent among individuals with the rs6242 AA genotype for vaspin. OSA was more severe in individuals with the AA genotype. Oxidative stress and endothelial dysfunction caused by this condition may lead to more critical conditions in OSA such as coronary artery disease, diabetes mellitus, and cerebrovascular diseases. Therefore, vaspin rs6242 genotype analysis in OSA patients may help to closely monitor these patients in the early period and prevent possible comorbidities.
